# Pork as Food

**Published:** 1859-01

**Authors:** 


### PORK AS FOOD.

" A fat hog is the very quintessence of scrofula and carbonic acid gas, and he who eats it must not expect thereby to build up a sound physical organism. While it contributes heat, not the twentieth part of it is nitrogen, the base of muscle." One of our cotemporaries cordially endorses the above sentiment as being sound practical truth, and says—" Fat pork was never designed for human food. It is material for breath and nothing more. See Liebig, and other organic chemists and physiologists. It makes no red meat or muscle. The prize fighter is not allowed to eat it. All that is not consumed by the lungs, remains to clog the body with fat."

The above is an average specimen of the twaddle which finds its way into the newspapers, and when once there, like the man with a cork leg, it never stops. It sounds racy, and for the sound, lazy editors scissor it out, never taking the trouble to analyze a single statement, or to summons up one of the hundreds of facts which have come under their own observation, and prove the absurdity of the quotation.

Without any theorizing on the subject, let any man raised on a farm, go back to the home of his childhood, and deny, if he can, that any day ever passed in which some part of a pig was not placed on his father's table.

Go to Virginia, to Tennessee, to Kentucky, the very land of hog and hominy; or let any one go with us to our own native county of Bourbon, and we will point him to the parents and to their children, who coming to town to school, were the associates of our childhood, and if promenaded along Broadway, the point which would attract the chief attention would be their giant size. To be specific, we will mention names of families, almost all six footers and nearing two hundred pounds. Whoever saw a Bedford or a Clay, or a Breckenridge that was a runt? Henry and Cassius M., and the peerless " Bob," for example—men as large in heart as in person—men who never knew fear, and who in mental power and in natural eloquence rank among the foremost. There are also the Garrards, the Williams, the Spears, the Scotts, and going along the old "Lime Stone Road," we come in among the settlement of the Howards, measuring seven feet or more in their stockings; and not far from there were the Lyles, the "Infant John," measuring six and a half, perhaps, and perhaps more, with a "heft accordin."

Look at the article again: "A fat hog is the very quintessence of scrofula." Where is the proof? That a fat hog is made up of carbonic acid gas, as such, is a "whopper!" The biggest pig in a poke hasn't enough of carbonic acid gas in him when dead, to kill a cat. "Not one twentieth part of a pig is nitrogen." And suppose it is'nt: not any part of pure milk, worth naming, is nitrogen, "the base of muscle," while in corn starch, tapioca, sago, arrow root, and sugar, there is not a particle, and yet they are by common consent allowed to be most excellent and healthful articles of food. It is acknowledged that fat pork "contributes heat, and is material for breath, nothing more." What of that? Every man and woman we ever saw, needs both "heat and breath." The sugar on our tables, the very best of Stewart's syrup, are as much the "quintessence" of "heat and breath" as "fat pork." "Fat pork is the material for breath and nothing more"—just as grass butter is! "Fat pork makes no red meat or muscle." Where is the proof? Besides how much of the "base of muscle" is there in a cart load of butter. A ton of arrowroot and a barrel of sugar would'nt "make red meat" enough to make a dinner for a mouse.

The fact is we don't care what Liebig, or any other Dutchman "says." We would'nt give a button for the mere *ipse dixit* of any man. We must open our eyes and use our brains, if we have any, and be willing learners of actual whole facts, and go where they carry us. We do not believe there is a man of mind enough to have obscured the lesser lights around him, in all this land, of whom it may not be said in literal truth, that on an average, a day never passed that he did not at some one of the three daily meals eat some portion of a pig. But responds the pork eater, "That may be so, but the ill effect of pork eating has not had time to make itself felt in them, but that it is now manifesting itself in the decay of the present generation." But there is such an inconclusiveness in that kind of argument, that only a dolt would use it, especially when the assumption of the fact that the present generation is in a state of decay, is made in the face of a known truth, that the average length of human life is greater now than it has been in a thousand years. Hence we come back to the sentiment often expressed in this journal, that all the good things of this life were given us by a Loving Father, richly to enjoy, in moderation and thankfulness; the proof of the "goodness" ot any thing being in the fact that whole communities have used it daily for generations, leaving sons stalwart in body, peerless in mind, with daughters as pure as the dew drop, and beautiful as the morning.

